# Emerging mechanisms in dry eye disease: apoptosis-associated tear MicroRNAs and toxicant-related alterations of the glycocalyx and lipid rafts

**DOI:** 10.3389/fopht.2026.1924390

**Published:** 2026-09-17

**Authors:** Mazyar Yazdani

**Affiliations:** Department of Medical Biochemistry, Oslo University Hospital, Rikshospitalet, Oslo, Norway

**Keywords:** apoptosis, dry eye disease, environmental toxicants, glycocalyx alterations, lipid raft disruption, tear microRNAs, haptenation, nanoparticles

## Abstract

**Background:**

Dry eye disease (DED), a multifactorial condition of the ocular surface, is marked by disruption of tear film homeostasis that can result in ocular discomfort, visual impairment, and potential epithelial damage. Its high prevalence and impact on quality of life and healthcare utilization underscore the need to better understand its underlying mechanisms. Although current research has clarified several key contributors, including tear film instability, ocular surface inflammation, and neural dysfunction, the complex pathophysiology of DED remains only partly understood. Moreover, new mechanistic insights are needed to advance diagnostic and therapeutic approaches.

**Hypothesis:**

This article highlights three emerging and underexplored mechanisms that may contribute to DED, based on a combination of disease-associated observations, indirect evidence, and mechanistic inference: (i) apoptosis-related tear microRNAs (miRNAs) as potential regulators of epithelial damage, (ii) ocular surface glycocalyx alterations potentially associated with environmental pollutants, and (iii) lipid raft disruption as a plausible mediator of toxicant-induced responses in ocular surface cells.

**Supporting evidence:**

First, apoptosis-related miRNAs in tears, traditionally viewed as potential biomarkers of the disease, are discussed as candidate regulators of epithelial damage through modulation of apoptotic pathways, although direct evidence in DED causation/development remains limited. Second, glycocalyx alterations potentially associated with environmental pollutants, such as airborne nanoparticles and reactive volatile compounds, are considered. This consideration draws in part on findings from non-ocular and non-DED systems. Third, lipid raft disruption is presented as a plausible but as yet untested mechanism in the context of the ocular surface and DED, potentially linking toxicant exposure to downstream cellular responses.

**Conclusion:**

By distinguishing between established knowledge, disease-associated findings, and forward-looking hypotheses, this work aims to broaden current perspectives on DED pathophysiology and suggest novel directions for future research.

## 1 Introduction

Dry eye disease (DED) is a multifactorial condition of the ocular surface characterized by loss of tear film homeostasis. It can cause ocular discomfort, impair visual function, and in some cases, lead to ocular surface damage. The disease affects a substantial proportion of the global population and is increasingly regarded as a public health issue because of its negative impact on quality of life, work productivity, and healthcare utilization (1, 2).

Elucidating the underlying mechanisms of DED is essential for advancing its prognosis, diagnosis, monitoring of disease progression, and therapeutic strategies. Although research over the past decades has provided valuable insights, the intricate pathophysiology of DED remains incompletely understood due to its multifactorial nature (3, 4). Tear film instability, ocular surface inflammation, and neural dysfunction are recognized key contributors to the disease (5). However, much remains to be discovered, and emerging studies are beginning to reveal additional mechanisms and potential therapeutic targets (6).

Apoptosis, a form of programmed cell death, plays a critical role in maintaining ocular surface homeostasis. However, excessive or dysregulated apoptosis can exacerbate DED symptoms by disrupting the epithelial barrier, enhancing inflammatory responses, and sustaining a vicious circle of cellular damage and death (7). Among studies investigating the underlying mechanisms of DED, tear microRNAs (miRNAs) have not previously been discussed in the context of apoptotic regulation. Although these regulatory RNAs have attracted increasing attention in recent years within the DED field, their relevance has largely been confined to potential use as biomarkers (6, 8). Given the important roles of miRNAs in various cellular processes, particularly in the regulation of apoptosis (9), these small non-coding RNAs may plausibly modulate apoptotic pathways; however, this represents a mechanistic hypothesis that is largely extrapolated from non-DED and non-ocular systems and requires direct validation.

The glycocalyx, a sugar-rich structure covering the apical side of ocular epithelial cells, is recognized as one of key determinants of tear film stability and ocular surface protection, and its compromise is considered an initiating event in the pathogenesis of DED (8, 10). Although recent studies have begun to uncover mechanisms linking glycocalyx alterations to DED (6), our knowledge is still evolving. One area of particular interest is the effects of environmental pollutants due to their presence in both air (11, 12) and the tear film (13, 14). For example, mechanistic insights into the interactions between airborne nanoparticles and the ocular surface glycocalyx that may compromise its integrity and function are limited. Additionally, nanoparticles can influence the expression of membrane-associated mucins in glycoproteins, which serve as primary structural anchors for the glycocalyx sugar (15). Importantly, much of the current understanding of pollutant–glycocalyx interactions is derived from broader glycocalyx literature outside the ocular surface context, and direct evidence in DED remains scarce. To date, no studies have examined whether toxicants like formaldehyde, a volatile organic compound in indoor and outdoor air, can induce haptenation through interactions with membrane-associated mucins or other glycocalyx-structuring proteins. Haptenation occurs when small reactive molecules bind covalently to endogenous or exogenous carriers, commonly proteins, creating altered antigenic sites that can trigger allergic reactions (16).

Lipid raft disruption is another largely understudied aspect of DED etiology despite some studies on individual membrane components and their associated signaling molecules and pathways. They are essential for key cellular processes, including signal transduction and involvement in processes like inflammation and apoptosis. Accumulating data show that these microdomains act as central platforms through which environmental toxicants exert their effects, potentially initiating or amplifying toxic responses within cells (17). While some studies have examined lipid raft interactions with bacteria (18–20), their role in mediating the effects of toxicants on corneal and conjunctival epithelia, particularly in the context of DED, remains hypothetical and has not yet been directly investigated.

Rather than establishing a single linear relationship among these three mechanisms, this article highlights underexplored contributors that may independently or interactively contribute to DED pathophysiology. Several conceptual links among these mechanisms provide the basis for a broader framework. First, with respect to apoptosis, tear miRNAs are discussed as apoptosis-related mediators within the tear film, while both the glycocalyx and lipid rafts participate in apoptotic regulation through membrane-associated signaling pathways. Second, in the context of environmental stressors, tear miRNAs represent biologically derived agents, whereas microplastics and other pollutants are considered foreign apoptotic stressors; correspondingly, the glycocalyx and lipid rafts function as critical interfaces and signaling platforms for toxicant-induced cellular responses. Third, a unifying theme across all three is epithelial integrity: the tear film serves as a carrier of endogenous and exogenous agents, the glycocalyx acts as a structural and biochemical barrier as well as a signaling interface, and lipid rafts regulate membrane-associated signaling that ultimately shapes downstream responses such as survival or damage. Throughout this work, an effort is made to distinguish between established findings, disease-associated observations, mechanistic inferences, and forward-looking hypotheses, acknowledging the varying levels of evidence supporting each component.

In light of these understudied yet potentially important mechanisms, the present work aims to highlight three emerging areas that may contribute to the pathophysiology of DED but have received limited attention to date: (i) apoptosis-related miRNAs in tears as potential regulators of epithelial damage, (ii) glycocalyx alterations with emphasis on potential pollutant-driven structural and functional compromise, informed partly by indirect evidence, and (iii) lipid raft disruption as a plausible mediator of toxicant-induced cellular responses (**Figure 1**). By bringing these mechanisms together, this article aims to expand the current understanding of DED beyond established pathways and to propose conceptual models for testing in future research.

**Figure 1:**
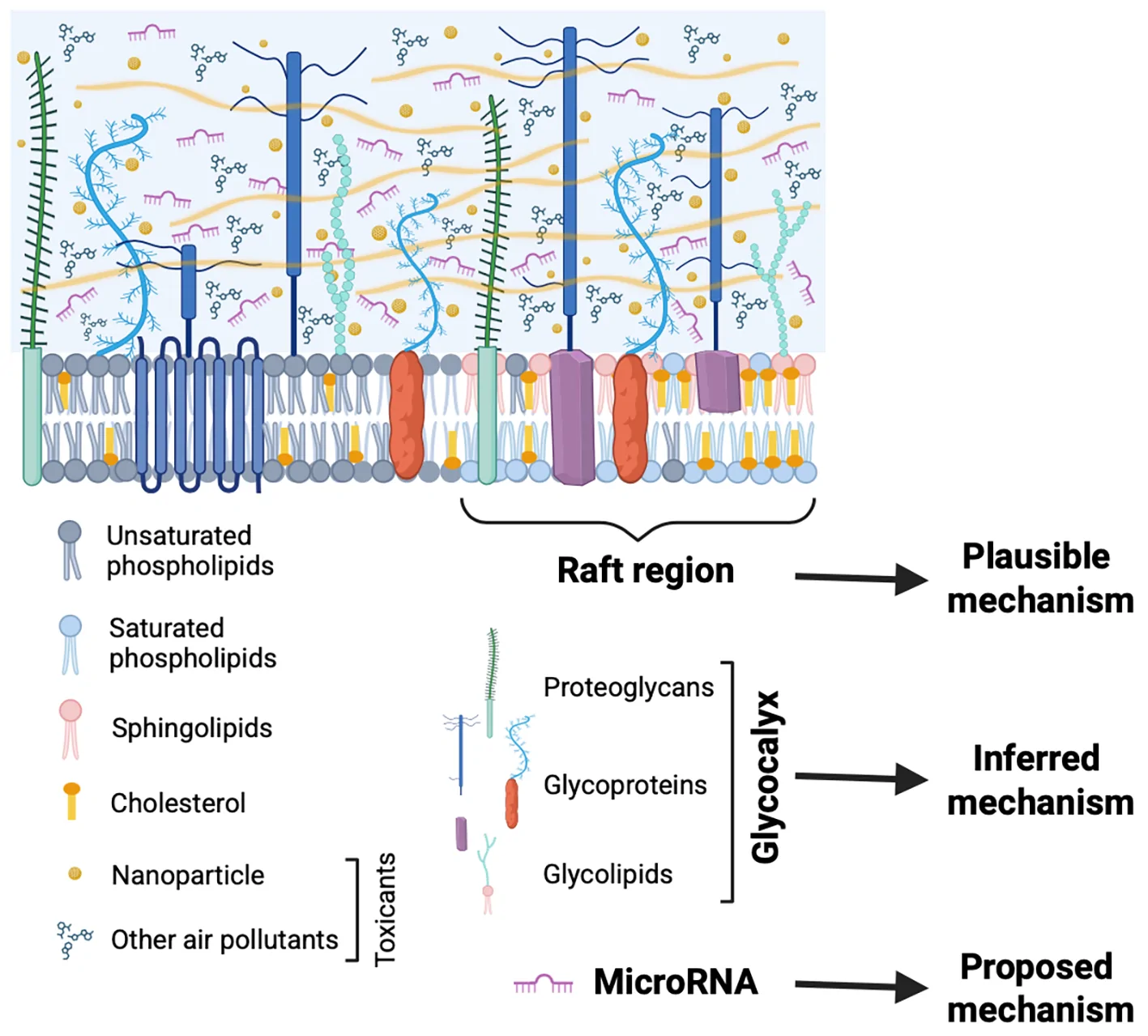
Schematic overview illustrating three emerging mechanisms potentially relevant to the pathophysiology of dry eye disease: (i) apoptosis-related microRNAs in tears as a proposed mechanism; (ii) ocular surface glycocalyx alterations potentially associated with environmental toxicants (e.g., nanoparticles) as an inferred mechanism; and (iii) lipid raft disruption as a plausible mediator of toxicant-induced responses in ocular surface cells. The image is intended to highlight conceptual relationships rather than established mechanistic pathways. Created with BioRender.com.

## 2 Known mechanisms

According to the Tear Film and Ocular Surface Society (TFOS) Dry Eye Workshop (DEWS) II Pathophysiology Report (5), DED arises from a loss of tear-film homeostasis in which tear film instability and hyperosmolarity play central roles. Regardless of the initial trigger, environmental and/or physiological factors, the disease is sustained by a self-perpetuating vicious circle involving hyperosmolarity, tear film instability, and ocular surface inflammation. This drives a persistent immune response, which is well documented in patients with DED (21).

In addition to the immunoinflammatory pathophysiology, involving both innate and adaptive immunity (22), ongoing research continues to explore other mechanisms underlying DED. For example, oxidative stress has emerged as a key driver and a potential link to central pathological processes of the disease (23, 24). Along these lines, oxidative stress-mediated ferroptosis has recently been implicated in DED pathogenesis (25). Moreover, in light of the proposed role of the tear film lipid layer in corneal oxygenation (26), oxidative stress arising from perturbations in it underscores the multifaceted contributions of this pathway to DED pathology. Oxidative stress has also been linked to the ocular surface microenvironment (27), a concept introduced in 2017 suggesting that DED pathogenesis may involve a complex interplay among resident microorganisms, locally produced metabolites, and coordinated immune responses (28).

The TFOS DEWS III Report expands upon DEWS II by emphasizing the growing complexity of DED and the interplay of multiple contributing mechanisms (8). Rather than relying on a strict binary distinction between aqueous-deficient (ADDE) and evaporative (EDE) forms, DEWS III adopts a driver-based framework that highlights etiological factors, such as tear film abnormalities, eyelid and adnexal dysfunction, and ocular surface changes, to support more personalized management strategies. Evaporative drivers commonly arise from alterations at the ocular surface and adnexa, such as meibomian gland keratinization, reduced blink efficiency associated with prolonged visual tasks and lid-related abnormalities. These factors destabilize the tear film, thereby increasing evaporation and tear film breakup, which in turn elevate osmolarity and trigger downstream inflammatory pathways and epithelial changes. In early or milder stages, these processes may present with more localized signs; however, as the disease progresses, inflammation becomes an integral component of the disease. In contrast, aqueous-deficient drivers are often linked to impaired lacrimal gland function, frequently associated with autoimmune conditions or hormonal influences, and may exhibit a more prominent inflammatory component from earlier stages. This inflammatory milieu drives cascades of proteases, cytokines, and T-cell-mediated responses, thereby sustaining the self-perpetuating vicious circle characteristic of DED (8).

## 3 Emerging mechanisms

In addition to the established mechanisms of DED, this section introduces several understudied and unexplored hypotheses that may contribute to disease onset and progression. Extending beyond the classic view, these concepts have the potential to provide fresh insights into disease pathophysiology and to stimulate further investigation into processes that remain poorly understood.

Because several of the proposed mechanisms remain insufficiently investigated at the ocular surface, the level of supporting evidence differs among the three topics discussed. Where direct ocular or DED-specific evidence is unavailable, the hypotheses presented are explicitly informed by mechanistic findings from non-ocular systems and should therefore be regarded as conceptual frameworks requiring experimental validation in ocular models.

### 3.1 Apoptosis-inducing factors in tears

The tear film, which maintains optimal conditions for the ocular surface, is continuously exposed to external foreign agents as well as circulating molecules from within the body. Some of these can trigger apoptosis in ocular surface cells. Tear miRNAs, as endogenous examples, have gained attention in DED since the beginning of this decade; however, their potential role in regulating apoptosis associated with the disease has not yet been addressed.

#### 3.1.1 microRNAs

The miRNAs are a class of short (18–22 nucleotides), non-coding RNAs that act as key regulators of post-transcriptional gene expression (29). It is estimated that humans have over 2,700 miRNA sequences (30), with over 60% of all protein-coding genes predicted to be potential miRNA targets (31). Beyond their intracellular roles, miRNAs are released into the extracellular environment, where they contribute to cell–cell communication and are associated with various biological and disease mechanisms (32). Notably, they are detectable in biofluids (33) and exhibit remarkable stability, maintaining integrity even after prolonged storage and repeated temperature fluctuations (34). These properties highlight their potential utility as biomarkers and candidates for clinical applications (35).

There has been increasing interest in studying miRNAs in ocular diseases (33, 36, 37). Despite containing notable levels of miRNAs – e.g., more species than serum and aqueous humor in patients with ocular disease (38) – the tear film has historically been underexplored due to the difficulty of obtaining sufficient tear volumes for comprehensive characterization (39). This remains a key challenge in DED research, as tear volume varies considerably among patients depending on disease stage (normal, mild, moderate, or severe) as compared with healthy individuals, resulting in a limited number of reports that have emerged relatively late. To address this issue, pooling tears from both eyes (39) and instillation of 2.5 µL of distilled water prior to sample collection (40) have been employed; however, further studies are needed to evaluate the impact of inter-eye variability and the potential dilution effects introduced by these approaches. The use of *in vitro* systems, such as cultured corneal epithelium (41), conjunctival epithelium (42) and ocular surface models incorporating a tear film system (43), provides a valuable alternative for mechanistic studies. However, these systems have not yet been applied to investigate miRNAs in DED.

**Table 1** summarizes the miRNA reports on patients with DED and Sjögren syndrome in association with the ocular surface. Two of the DED studies compared the expression profile of tear miRNAs between patients and controls (39, 44), whereas the third one compared profiles between two subgroups of DED patients based on tear film stability (45). Regarding Sjögren syndrome, a DED-associated condition, two studies focused on patient-control comparative profiles (40); one examining 43 different tear miRNAs and the other investigating altered miRNA expression patterns in collected conjunctival epithelial cells (46). Two other Sjögren syndrome studies evaluated two specific miRNAs each in tears (47) and peripheral blood mononuclear cells (48). The differential expression profile of miRNAs in patients has therefore proposed as a potential molecular biomarker for disease diagnosis. In addition to human studies, a few attempts to find potential biomarkers were made by investigating miRNA expression levels in lacrimal glands (49), serum (50) and tears (51, 52) from NOD mice, a Sjögren syndrome model exhibiting a dry eye manifestations. The differentially expressed miRNAs, especially in patients (**Table 1**), were suggested to be associated with immune and inflammatory pathways. Accordingly, in the absence of direct functional evidence demonstrating apoptosis regulation by tear miRNAs in ocular surface cells, the present proposal is primarily based on indirect data and requires experimental validation. Importantly, as summarized in **Table 1**, none of the published patient-based studies has functionally validated whether the differentially expressed tear miRNAs directly regulate apoptosis in corneal or conjunctival epithelial cells. Consequently, current evidence remains largely associative, linking altered miRNA expression to DED, inflammation, or immune-related pathways without demonstrating a causal role in ocular surface apoptosis. This represents one of the principal knowledge gaps underlying the present hypothesis and highlights the need for functional validation in ocular surface models.

**Table 1:** Summary of patient-based miRNA studies in dry eye disease (DED) and Sjögren syndrome (SS) reported ophthalmic evaluations.

Disease type	Ocular assessments	Sample size (n)	Sample type	Differential expression	Suggested role	Apoptosis functional validation in ocular surface cells (yes/no)	Reference
Dry eye							
1	Recruited patients with dry eye-related symptoms and signs	138 patients, 138 healthy controls	Tear wash and capillary collection	4 upregulated: 450b-5p, miR-1283, miR-5700, miR-3671 28 downregulated: miR-4673, miR-890, miR-576-5p, miR-337-3p, miR-607, miR-1827, miR-485-5p, miR-6884-5p, miR-335-3p, miR-647, miR-4433b-5p, miR-570-3p, miR-1276, miR-2054, miR-802, miR-539-5p, miR-198, miR-3646, miR-4427, miR-4705, miR-606, miR-20b-3p, miR-4475, miR-4478, miR-4797-5p, miR-142-5p, miR-153-5p, miR-5583-5p	Linked to inflammation	No	Wang et al. (2020) (44)
2	Recruited patients with dry eye-related symptoms and signs	5 patients, 5 non-DED	Tear wash and capillary collection of both eyes	9 upregulated: miR-127-5p, miR-1273h-3p, miR-1288-5p, miR-130b-5p, miR-139-3p, miR-1910-5p, miR-203b-5p, miR-22-5p, miR-4632-3p	Associated with inflammatory pathways	No	Pucker et al. (2022) (39)
3	Recruited patients with dry eye-related symptoms	Group 1 with TFBUT 2s (M: 2, F: 3), group 2 with TFBUT 10s (M:3, F:2)	Tear collected by Schirmer strip and kept in PBS	Identified 34 immature tear miRNAs between TFBUT 2s and 10s groups. 15 had signal values ≥20 and a fold change ≥1.5: miR-6855, miR-4740, miR-4284, miR-1301, miR-130B, miR-6720, miR-4428, miR-3132, miR-4499, miR-6815, miR-1227, miR-6892, miR-125A, miR-4722, miR-933	Unclear biological roles in recipient cells	No	Cross et al. (2023) (45)
Sjögren							
1	Evaluated visual analog scale (VAS) for dry eye	27 patients (M: 2, F: 25), 22 healthy controls	Peripheral blood mononuclear cells	Upregulated miR-146a and downregulated miR155, both positively correlated with VAS	Involved in immune regulation	No	Shi et al. (2014) (48)
2	Recruited patients with dry eye-related signs	18 patients (M: 2, F: 16), 8 controls	Tear collection using micropipettes In the case of few tears, same method used after instillation of 2.5 µL of DW	4 upregulated: miR-16-5p, miR-34a-5p, miR-142-3p, miR-223-3p 10 downregulated: miR-30b-5p, miR-30c-5p, miR-30d-5p, miR-92a-3p, miR-134-5p, miR-137, miR-302d-5p, miR-365b-3p, miR-374c-5p, miR-487b-3p Non significantly correlated with the OSS	Related to autoimmunity and neuropathy	No	Kim et al. (2019) (40)
3	Recruited patients with dry eye-related symptoms and signs	20 patients, 20 healthy; both M and F included	Tear wash and capillary collection	Upregulated miRNAs 155, but not miR-181	Linked to immune and inflammatory responses	No	Aragón-Arreola et al. (2021) (47)
4	Recruited patients with dry eye-related symptoms and signs	6 patients (M: 0, F: 6), 6 healthy controls (M: 0, F: 6),	Conjunctival epithelial cells from the upper and lower fornixes using nylon-tipped swab	10 upregulated: miR-1245b-3p, miR-219b-3p, miR-431-5p, miR-4787-3p, miR-491-3p, miR-5010-5p, miR-519b-5p, miR-548a-5p, miR-548ai 1 downregulated: miR-548j-3p	associated with immune and inflammatory regulation	No	Giovannetti et al. (2025) (46)

*DW, distilled water; F, female; M, male; OSS, Ocular staining score; PBS, phosphate-buffered saline; SS, Sjögren syndrome; TFBUT, tear film break-up time.

While the present hypothesis proposes that tear miRNAs may actively regulate apoptosis in DED, an alternative interpretation is that the altered tear miRNA profiles primarily reflect ongoing epithelial injury, inflammation, or tear film instability rather than functioning as direct mediators of disease. Under this view, tear miRNAs may serve principally as biomarkers of disease activity or compensatory responses rather than causal regulators of disease progression. These interpretations are not necessarily mutually exclusive, however, as specific miRNAs may function both as indicators of pathological processes and as active modulators of apoptotic signaling. Distinguishing between these possibilities will require functional validation in ocular surface models.

MiRNAs play important roles in various cellular processes, particularly in regulating apoptosis, which is a form of programmed cell death essential for maintaining cellular homeostasis and eliminating damaged or unwanted cells (9). Interestingly, several of the tear miRNAs identified in the above studies have been linked to apoptosis in other biological contexts, such as miR-127-5p (53), miR-450b-5p (54), miR-22 (55), miR-1283 (56), miR-16-5p (57), miR-34a-5p (58), miR-142-3p (59), miR-223-3p (60), miR-30b-5p (61), miR-374c-5p (62), miR-337-3p (63), miR-335-3p (64), miR-539-5p (65), miR-142-5p (66), miR-431-5p (67) and miR-491-3p (68). Although apoptosis-related roles of miRNAs have been described in certain ophthalmic research (33, 36), the potential contribution of tear-derived miRNAs to apoptotic processes in DED has not yet been explored in the literature.

Apoptosis occurs through two primary pathways: the intrinsic (mitochondrial) and the extrinsic pathways. The former is activated by intracellular stress signals such as DNA damage, cytotoxic drugs, growth factor withdrawal, or oxidative stress. The latter is initiated by external signals from other cells or the extracellular environment within an organism. Crosstalk between the two pathways occurs through shared signaling intermediates, integrating them into common downstream mechanisms. Beyond these, prolonged endoplasmic reticulum stress can also induce apoptosis, and its signaling cascades may converge with those pathways. MiRNAs play an important regulatory role in apoptosis and many are involved in these pathways. Depending on the cellular context and their targets, miRNAs can function as either pro- or anti-apoptotic molecules. They influence gene expression directly, by binding to messenger RNAs (mRNAs), or indirectly, by modulating the activity of other miRNAs (9, 69). Therefore, miRNAs are involved in nearly all phases of apoptosis, can be used for diagnosis and treatment in a wide range of diseases, and could contribute to disease prevention due to their role in pathogenic processes (70).

Functionally, miRNAs guide the miRNA-RNA-induced silencing complex (miRNA-RISC) to regulate target mRNAs, typically binding to 3′-UTRs through Watson-Crick pairing in the seed region (nucleotides 2–8). The degree of complementarity determines the outcome: perfect matches induce Argonaute (Ago)-mediated mRNA cleavage, while central mismatches block cleavage but suppress translation (69). The influence of miRNAs is not exclusively inhibitory. For example, both their negative and positive effects on the cornea and lacrimal gland have been demonstrated previously (37). With limited reports available, it remains unclear whether miRNAs originate from stressed cells during the vicious circle of DED development and progression through established mechanisms, or whether they result from an alternative independent pathway, or a combination of both.

Evidence from a few studies provides some support for the former idea, particularly via miRNAs’ association with inflammation and oxidative stress. Pucker et al. (39) have linked the profile of differentially expressed miRNAs in tears from DED patients to inflammation using pathway analysis. Moreover, some miRNAs in the tears of patients at the interface between ocular hypertension and primary open-angle glaucoma, along with their target genes and associated signaling pathways, have been shown to be linked to apoptosis, inflammation, and oxidative stress (71). Similarly, a study on plasma and tear samples from diabetic retinopathy patients demonstrated that oxidative stress mediates epigenetic modifications and the expression of tear miRNAs and apoptosis-related genes (72).

Alternative independent pathways may involve the distal transport of miRNAs through biofluids. This process primarily occurs via two mechanisms: active transport within extracellular vesicles (EVs) and carriage in protein-miRNA complexes. In addition, a part of circulating miRNAs may originate from ruptured or injured cells. Once secreted, particularly those encapsulated in EVs (mechanisms reviewed in Chopra et al. (73)), these miRNAs can facilitate paracrine or endocrine signaling across different tissues, thereby influencing gene regulation and cellular activity at distant sites (74). To date, Pucker et al. (39) and Cross et al. (45) have investigated the miRNA content of EVs isolated from the tear fluid of DED patients. However, the diverse origins of tear components, arising from both proximal (e.g., corneal epithelial and conjunctival goblet cells) and distal sources (e.g., lacrimal and meibomian glands) (45), complicate data interpretation and call for further studies. Moreover, emerging evidence linking tear miRNAs to other ocular (e.g., glaucoma and diabetic retinopathy) and non-ocular diseases (e.g., Alzheimer’s disease and breast cancer) (33) highlights the need for additional research to elucidate their biological significance and potential roles in intercellular communication and systemic signaling. Recent studies in glaucoma further support the translational potential of ocular miRNAs as clinically relevant biomarkers, reinforcing their broader applicability across ophthalmic diseases beyond DED (75).

It is also important to investigate the influence of exogenous agents and other endogenous factors in triggering apoptosis. Among the exogenous agents, microplastics represent a notable example, as humans are frequently exposed to them through air, water, food (76), and medical devices (77). As a result, the ocular surface, like other organs, is exposed, which can lead to conditions such as DED (78, 79). Polyethylene, the predominant type detected in tear fluid and meibum, showed significant correlations with key DED parameters, and *in vitro* exposure reduced cell viability and induced apoptosis in human corneal and conjunctival epithelial cells in a dose-dependent manner (80). With respect to endogenous factors, studies have shown that, in addition to miRNAs, various RNA subtypes including mRNA, long non-coding RNA (lncRNAs), circular RNA (circRNA), small nuclear RNA (snRNA), small nucleolar RNA (snoRNA), transfer RNA (tRNA), small Cajal body-specific RNA (scaRNA), Y RNA (yRNA), and ribosomal RNA (rRNA) are present in the tears of DED patients (45). Interestingly, it has been suggested that fragmented forms of lncRNA, snRNA, and tRNA may function similarly to miRNAs, while lncRNA, circRNA, and mRNA can contain sequences that compete for miRNA binding, effectively acting as miRNA sponges (81). This points to a complex network of interactions among different RNA subtypes, in both full-length and fragmented forms, underscoring the intricate regulatory potential of tear fluid RNAs (45). Therefore, investigating their specific roles in apoptosis—and in ocular physiology and pathology more broadly—is particularly compelling. Apart from these, an equally intriguing area of research concerns the involvement of miRNAs in autophagy and necroptosis (programmed necrosis), the other major forms of programmed cell death (69).

Progress in understanding the functional role of tear miRNAs in DED is constrained by several methodological and conceptual challenges. These include limited tear volume and variability in sampling approaches, uncertainty about the cellular origin and stability of extracellular miRNAs, and the lack of experimental systems that accurately model their extracellular delivery and uptake by ocular surface cells. In addition, most current evidence is associative, with little direct validation of apoptosis-related targets in ocular tissues. Addressing these limitations will require standardized tear collection and processing protocols, coupled with approaches that distinguish free miRNAs from those encapsulated in extracellular vesicles. Functional studies may be advanced by corneal and conjunctival epithelial models exposed to patient-derived tear fractions or isolated miRNA populations, combined with apoptosis assays and target validation strategies such as reporter-based systems. Integration of transcriptomic and proteomic datasets may further help define miRNA-mediated regulatory networks relevant to epithelial damage in DED.

### 3.2 Cell membrane-associated mechanisms

The ocular surface is lined by two continuous epithelial tissues: the corneal and conjunctival epithelia. The former is essential for preserving corneal transparency, structural integrity, and avascularity. The latter extends from the eyelid margin to the limbus, covering the inner eyelids and the exposed scleral surface, and contains mucin-secreting goblet cells. Immune cells and fine nerve endings are embedded within these layers (82, 83). The corneal and conjunctival epithelia exhibit apical-basal polarity (84), which is manifested by two distinct plasma membrane domains: the apical surface covered by glycocalyx, exposed to the external environment, and the basolateral surface, facing adjacent and underlying cells. These domains differ markedly in composition and organization, each fulfilling specific functions (85). Perturbations in these membrane-associated structures and interactions may contribute to the pathogenesis of DED.

#### 3.2.1 Glycocalyx alterations

The apical surface of the ocular epithelial cells features microplicae, membrane folds that increase the cell surface area. These folds are coated by a glycocalyx, a dynamic carbohydrate-rich structure that forms the interface between the epithelium and the tear film. The glycocalyx layer is composed of diverse sugar-based conjugates such as glycoproteins, proteoglycans, and glycolipids (**Figure 1**). Key functions of this layer includes protecting the epithelium from mechanical and chemical stress, contributing to cell signaling, limiting microbial access, reducing friction during blinking, and supporting surface wettability (86).

The primary structural anchoring of the carbohydrate is provided by transmembrane glycoproteins, notably membrane-associated mucins, which are the most widely studied and represent the largest components of the ocular surface glycocalyx. In humans, six mucin family members, namely MUC1, MUC4, MUC16, MUC20, MUC21, and MUC22, are known to contribute to the ocular surface glycocalyx. Glycans conjugate to proteins through several mechanisms, including 1) N-linkage to asparagine, 2) O-linkage to serine, threonine, or tyrosine, 3) C-linkage to tryptophan, and 4) via glypiation. Their attachment and final structure are determined by the glycogene repertoire of each cell, leading to tissue- and cell-specific glycosylation patterns. This results in distinct glycan profiles across the conjunctiva, cornea, and tear film, although the functional implications remain unclear (15, 87).

Reports on the ocular surface glycocalyx in DED have primarily examined changes in the expression of membrane-bound mucins and alterations in their polysaccharide chains. For the former, findings have been inconsistent in both Sjögren and non-Sjögren dry eye (15, 88). For example, while one study reported reduced conjunctival MUC1, MUC2, MUC4, and MUC5AC in non-Sjögren dry eye (89), others found no change in MUC1 or MUC16 (90, 91). More recent work showed increased MUC1, MUC4, and MUC16 in different conjunctival regions (92). For alterations in mucin glycosylation, reductions in several ocular surface glycans, such as sialic acid, N-acetyl-glucosamine, N-acetyl-galactosamine, and galactose–N-acetyl-galactosamine, have been reported in dry eye (93). The O-acetylated sialic acid epitope of MUC16 shows a disrupted pattern in non-Sjögren dry eye, replacing the normal mosaic appearance with a “starry sky” pattern (94, 95). Sialylated MUC1 epitopes are increased in mild–moderate disease but reduced in severe DED (96). In addition to mucins and their associated glycans, tear galectin-3, a glycan-binding protein that binds specific sugar moieties on glycoproteins, is also elevated in dry eye (97). Its elevation in tears is suggested to amplify interleukin-1β (IL-1β)–mediated inflammation, creating a self-reinforcing cycle that further destabilizes the glycocalyx (10).

In DED, factors that disturb tear homeostasis and trigger inflammation affect the ocular surface (6), including corneal and conjunctival epithelial cells and their glycocalyx composition (15). Owing to the limited research in this area, current understanding is largely derived from studies of other cell types. Consequently, the mechanistic pathways discussed below should not be interpreted as established mechanisms operating at the ocular surface. Rather, they represent biologically plausible concepts extrapolated primarily from vascular and other epithelial systems that warrant direct investigation in ocular tissues.

For example, research on the vascular endothelial glycocalyx is more developed and provides detailed insights into factors that compromise its integrity, such as inflammatory mediators, oxidative stress, ischemia-reperfusion, lipids, elevated glucose, high sodium, and female sex hormones, among other mechanisms promoting glycocalyx degradation and shedding (98). Although many of these are already well established for dry eye, only elevated glucose is not yet fully characterized in the disease. In addition, to clarify, sodium is already linked to the well-known tear hyperosmolarity driven largely by increased sodium ion concentrations (99). For elevated glucose, increased levels have previously been reported in tear metabolomics studies of DED patients by a Chinese research team (100) and more recently by our group (101). In line with this, recent meta-analytic evidence indicates that diabetes mellitus is associated with a significantly increased risk of DED, with hyperglycemia-related mechanisms such as oxidative stress, inflammation, and neuropathy contributing to tear film dysfunction and ocular surface vulnerability (102). The proposed mechanisms for glucose-induced glycocalyx damage are largely inferred from non-ocular tissues, particularly vascular and glomerular endothelium. These mechanisms include the generation of reactive oxygen species (ROS) due to mitochondrial fragmentation or cytoplasmic ROS under hyperglycemic conditions, the activation of glycocalyx-degrading enzymes, increased matrix metalloproteinase activity, increased heparanase levels, inactivation of the hyaluronic acid synthase 2 gene, and production of advanced glycation end-products (AGEs) leading to further damage to the glycocalyx (98).

A gap remains in the mechanistic understanding of interactions between the ocular surface glycocalyx and materials that may compromise its integrity and function. One example is exogenous particulate contaminants, such as nanoparticles found in industrial and commercial products, eye cosmetics, and air pollution. The research to date has primarily focused on the ocular toxicity of certain manufactured nanoparticles (103–105) and the link between air pollution, including various particulate matters, and ocular surface disease like dry eye (11, 12, 106). Mechanistic studies investigating the toxicity of nanomaterials on the eye have recently been identified as an issue worthy of investigation (107). This highlights the need for a deeper understanding of the chemical principles governing glycocalyx structure and the subsequent reactions that may influence nanoparticle interactions.

The glycocalyx, as a negatively charged gel, plays a central role in regulating ocular surface permeability. Chemically, compounds such as carboxylate and sulfate groups in glycosaminoglycans (proteoglycans) (108) and sialic acid (glycoproteins and glycolipids) (109) at the tips of glycans provide highly negative charges that contribute to their self-repelling properties, thereby stabilizing the bottle-brush arrangement of the glycans (109). These charges also render the glycocalyx surface highly hydrophilic (109), allowing it to attract and retain water (108). To access receptors and the cell membranes, nanoparticles must first interface with the cell surface glycocalyx, a mechanism that remains poorly understood, with most available insights coming from studies in nanomedicine and materials science (110).

The characteristics of the cell-surface glycocalyx and the properties of nanoparticles both influence their interplay. In general, charge-based repulsion and attraction within the glycocalyx determine which molecules can pass through or interact with it. This is based on indirect evidence from other tissues, which may help elucidate such interactions in ocular surface cells. For instance, the negatively charged barrier of the vascular endothelial glycocalyx restricts the passage of cationic molecules from the vasculature through electrostatic interactions (111), while anionic nanoparticles are typically repelled by the glycocalyx (112). This exclusion appears to depend on the degree of maturity, as anionic nanoparticles can be internalized by endothelial cells with a developing glycocalyx, but not by those with a fully mature glycocalyx (112). The nonspecific character of electrostatic effects may not apply uniformly to all nanoparticles or cell types, as these interactions can be influenced by structural features such as thickness and density. Furthermore, the glycocalyx can extend tens to hundreds of nanometers from the cell surface, and this outward length can sterically hinder nanoparticle–cell surface receptor engagement (110). The degree to which such interactions lead to binding, uptake, and particularly ocular cell toxicity is also influenced by nanoparticle properties, including their chemical components and surface chemistry, size and shape, surface area/charge and modification, dosage and concentration, and behavior in media (for review, see Zhu et al. (103)).

Toxic mechanisms of nanoparticles on the eye have been studied mainly from the perspective of light transmission and refraction, focusing on inflammation-driven corneal neovascularization, oxidative stress-induced corneal cell necrosis, and impaired ion pump function in corneal endothelial cells leading to corneal edema. Nanomaterial-induced conjunctivitis may arise through mechanisms indirectly inferred from those described for keratitis (107). Regarding glycocalyx composition, ophthalmic studies have remained above the nanoscale and have reported only mucin-related changes (106). Such alterations have revealed mechanistic insights in lung models, even though they involved a secreted, rather than membrane-associated, mucin. CuO nanoparticles, with diverse industrial applications, triggered mitogen-activated protein kinase (MAPK) signaling, driving both an inflammatory response and increased MUC5AC expression in healthy donor-derived airway epithelial cells and a murine asthmatic model (113, 114). These findings, obtained from respiratory tract models (non-ocular systems), therefore provide indirect support.

With widespread exposure to hazardous substances in indoor and outdoor air, the underlying mechanisms of ophthalmic toxicity, particularly at the ocular surface, have yet to be fully elucidated. One unexplored pathway is haptenation, in which low-molecular-weight (<1000 Daltons) chemicals covalently bind to endogenous or exogenous carriers, most commonly proteins, forming neoantigens and triggering an allergic response (16). For example, formaldehyde (30.03 Daltons), a volatile organic compound present in both indoor and outdoor air (115, 116), initially reacts with amine nucleophiles of certain amino acids to form Schiff-base adducts, with subsequent reactions generating more complex structures (117). Cell-based studies have suggested a potential association between this chemical and the ocular surface. In human corneal epithelial cells cultured under air–liquid conditions, formaldehyde has been shown to reduce cell viability, induce chemokine production, and trigger apoptosis, thereby contributing to ocular surface diseases such as DED and allergy (118). Consistently, formaldehyde gas exposure increases inflammation in an *in vitro* model of dry eye (119). A recent comprehensive database-driven in silico analysis exploring the molecular links between selected air pollutants (including formaldehyde) and dry eye identified six key genes, encompassing tumor necrosis factor (TNF), epidermal growth factor receptor (EGFR), matrix metalloproteinase-9 (MMP-9), estrogen receptor 1 (ESR-1), intercellular adhesion molecule-1 (ICAM-1), and matrix metalloproteinase-2 (MMP-2), as potential mediators of pollutant-induced onset and progression of DED (116).

Given the role of glycosylation in clinical allergy (120) and of the glycocalyx in ocular surface allergies (15), further research is warranted to clarify the potential contribution of the glycocalyx to allergic responses in relation to air pollutant exposure. Emerging data indicate that ocular allergy can contribute to dry eye (121), and more than half of affected individuals report concurrent DED (122, 123). However, until robust mechanistic studies establish a clearer link between allergic responses and DED, these findings remain suggestive.

Mechanistic insight into glycocalyx alterations at the ocular surface remains limited by the lack of methods to directly characterize glycocalyx structure and interactions at the nanoscale under physiological conditions. Much of the current understanding is extrapolated from non-ocular systems, particularly vascular endothelium, which may not fully capture the unique biochemical and biophysical properties of the ocular surface. In addition, experimental models that integrate environmental exposure with glycocalyx-specific outcomes are scarce. Future progress will depend on the application of advanced imaging and analytical techniques, including high-resolution and super-resolution microscopy, to visualize glycocalyx architecture and its perturbation. The development of *in vitro* ocular surface models, such as air–liquid interface systems, may provide controlled platforms for investigating interactions between pollutants and glycocalyx components. Complementary physicochemical approaches to model charge-based and steric interactions between nanoparticles and glycocalyx structures may further clarify the principles governing these interactions.

#### 3.2.2 Lipid raft disruption

The apical plasma membrane domain of corneal and conjunctival epithelia contains transmembrane mucins, for example, that contribute to the transcellular barrier, whereas the basolateral domain harbors junctional complexes that maintain the paracellular barrier (85). In addition, lipids are the principal structural components of epithelial cell membranes, and the mechanisms governing their polarized distribution are beginning to be elucidated (124). Compositional lipid microdomains, known as lipid rafts, within plasma membranes are believed to play key roles in various cellular processes (125). The term “lipid rafts” is often used as a broad designation encompassing various related structures, including rafts, detergent-resistant membranes, and caveolae (17). Despite ongoing debate regarding their existence and function *in vivo* (126), largely owing to their dynamic and nanoscale nature, increasing evidence supports the role of these microdomains in cell biology (127).

Lipid rafts are defined as dynamic plasma membrane microdomains that are detergent-resistant and highly enriched in cholesterol, glycosphingolipids, and phospholipids with saturated acyl chains (128). In epithelial cells, sphingolipids and phosphatidylinositol lipids in particular, play central roles in organizing the apical and basolateral plasma membrane domains (124). Since the concept of lipid rafts originally emerged from analyses of the lipid composition of apical membranes, it can be said that epithelial cells represent an optimal experimental model for investigating lipid functions (129). Lipid raft microdomains in corneal epithelial cells have previously been studied for bacterial interactions (18–20).

Lipid rafts function as platforms for signal transduction, participating in processes such as calcium and redox signaling, inflammation, immune regulation, hormone signaling, and cell death (e.g., apoptosis) (17). Growing evidence indicates that they serve as hubs mediating the cellular actions of both organic and inorganic environmental toxicants, contributing to the initiation or amplification of toxic cellular responses. The effects of these chemicals include alterations in raft organization and signaling, such as the accumulation of toxicants within lipid rafts, changes in lipid composition (including raft disruption, modifications of membrane lipids, and altered levels of raft-associated lipids), and modifications of the raft proteome (including changes in raft-associated proteins, protein aggregation within rafts, and activation of downstream signaling pathways) (17).

Although the lipid raft-mediated impact of toxicants has not yet been investigated in the corneal and conjunctival epithelia, this remains a hypothetical but potentially relevant mechanism, given the presence of air pollutants in tears (13, 14) and their documented effects on the ocular surface (115, 130–134), especially in relation to DED (135–138). The ocular surface is known to serve as an absorption route for toxicants entering the body (139), and tear fluid biomonitoring has been proposed as a promising approach for assessing environmental and chemical exposures (140). However, direct evidence linking lipid raft-mediated processes to toxicant effects in ocular surface epithelia is currently lacking and require studies in other models, particularly *in vitro* epithelial systems. Accordingly, the mechanistic concepts presented in this section are intended to generate experimentally testable hypotheses rather than to imply that equivalent lipid raft-mediated mechanisms have already been demonstrated in ocular surface biology or DED.

Indirect evidence from non-ocular systems and limited ocular contexts suggests that toxicants can affect ocular surface epithelia through lipid raft-mediated mechanisms. One example is the cystic fibrosis transmembrane conductance regulator (CFTR), a widely expressed apical chloride channel in epithelial tissues, which localizes to lipid-raft microdomains (18). Its presence in human corneal epithelial cells (19) and other epithelial cell systems (141) has been demonstrated through experiments showing its requirement for the internalization of *Pseudomonas aeruginosa*. Notably, these observations in corneal epithelial cells primarily relate to microbial interactions rather than toxicant exposure. Interestingly, exposure to secondhand smoke has been reported to reduce CFTR expression and disrupt raft-associated functions in a macrophage cell line (RAW264.7), providing a plausible explanation for the observed reduction in phagocytic capacity toward *P. aeruginosa* under such conditions (142). It remains to be established whether similar mechanisms operate in ocular surface epithelia under toxicant exposure, and this should be investigated in controlled *in vitro* studies.

The investigation of lipid raft involvement in DED is hindered by technical challenges associated with their small size, dynamic behavior, and sensitivity to experimental manipulation. Furthermore, there are currently no established ocular surface models that directly assess lipid raft–mediated responses to environmental toxicants, and most of the available evidence is derived from non-ocular systems. Advancing this field will require the adaptation of methodologies capable of probing membrane microdomains in living epithelial cells, including live-cell imaging and biochemical isolation of raft-associated fractions. Experimental approaches that modulate membrane lipid composition, such as cholesterol depletion or enrichment, may help clarify functional roles in signaling and apoptosis. Integration of such strategies with controlled toxicant exposure models in corneal and conjunctival epithelial systems could provide direct evidence for the relevance of lipid rafts in DED pathophysiology.

### 3.3 Mechanistic integration of the proposed mechanisms

The three mechanisms discussed in this article are presented individually for clarity; however, they are unlikely to operate as isolated biological processes. Rather, they may represent complementary components of a broader regulatory framework influencing ocular surface homeostasis through shared upstream stimuli and convergent downstream signaling pathways. Although direct experimental evidence linking these mechanisms in DED is currently lacking, the evidence and mechanistic considerations discussed herein provide a basis for several conceptual models (**Figure 2**). These models are not intended to propose a single linear pathogenic sequence but rather to illustrate how apoptosis-associated tear miRNAs, glycocalyx alterations, and lipid raft dynamics may independently or interactively contribute to DED pathophysiology.

**Figure 2:**
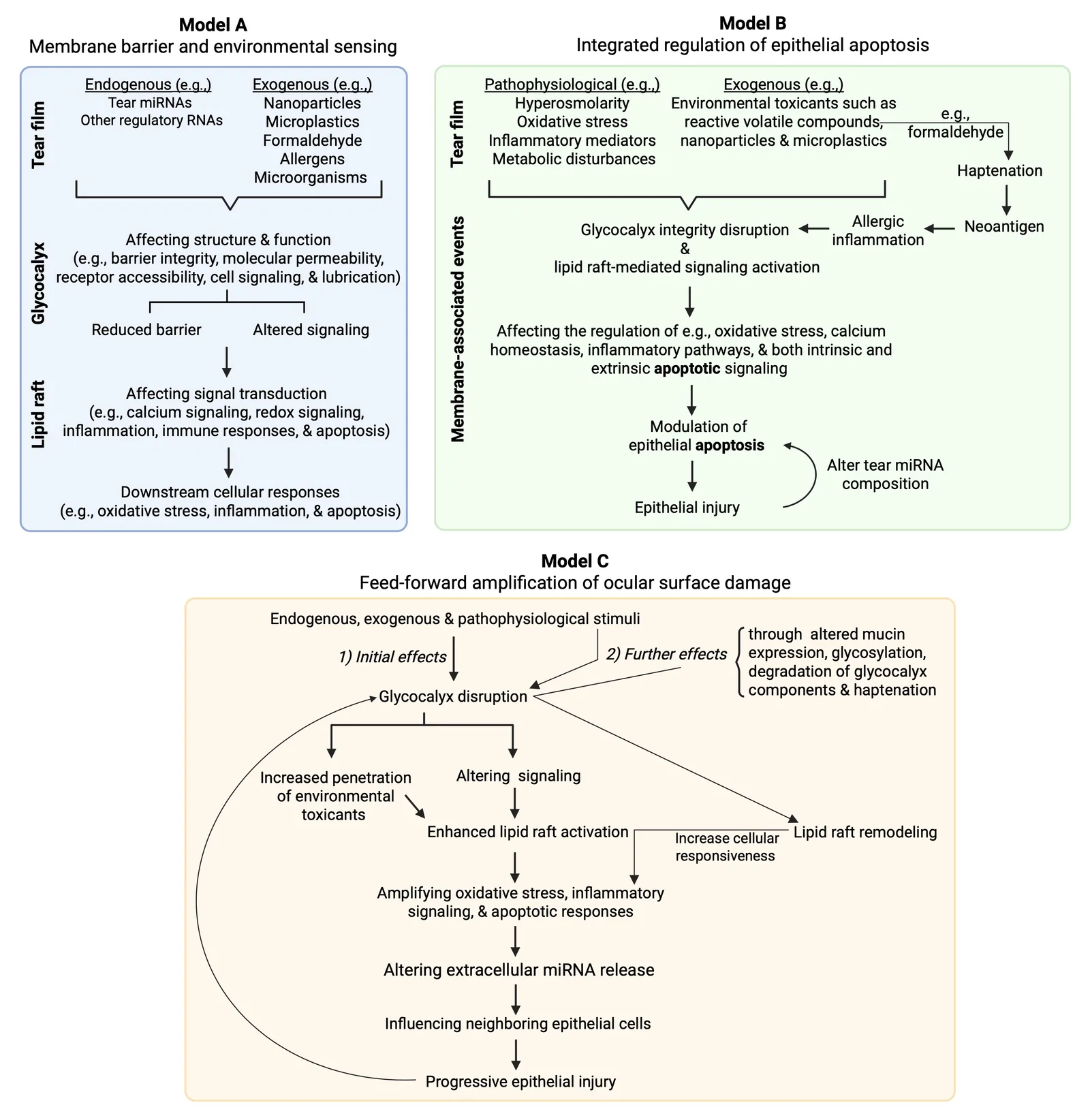
Three conceptual models illustrating potential mechanistic interactions among apoptosis-associated tear microRNAs, glycocalyx alterations, and lipid raft signaling in dry eye disease. Model A: Membrane barrier and environmental sensing; Model B: Integrated regulation of epithelial apoptosis; and Model C: Feed-forward amplification of ocular surface damage. The models represent conceptual hypotheses derived from the evidence and mechanistic considerations discussed in this article and are intended to illustrate potential mechanistic relationships rather than established pathogenic pathways. Created with BioRender.com.

#### 3.3.1 Membrane barrier and environmental sensing

The first model proposes that the tear film, glycocalyx, and epithelial plasma membrane constitute an integrated environmental sensing system rather than independent anatomical structures. The tear film continuously carries endogenous mediators, including apoptosis-associated miRNAs and other regulatory RNAs, together with exogenous agents such as nanoparticles, microplastics, reactive volatile compounds (e.g., formaldehyde), allergens, and microorganisms. Consequently, both endogenous and environmental factors may simultaneously influence epithelial homeostasis.

The ocular surface glycocalyx serves as the primary molecular interface encountered by these agents. Besides functioning as one of the principal determinants of tear film stability and epithelial protection, it regulates molecular permeability, receptor accessibility, cell signaling, lubrication, and interactions between epithelial cells and the external environment (15, 86, 109, 110). Structural alterations of membrane-associated mucins or their glycosylation may therefore compromise not only the barrier function of the glycocalyx but also its signaling properties. Likewise, nanoparticles must first interact with the glycocalyx before reaching membrane receptors, whereas reactive chemicals such as formaldehyde may induce haptenation through covalent modification of membrane-associated mucins or other glycocalyx-associated proteins, generating altered antigenic sites capable of initiating allergic and inflammatory responses (16).

Within this framework, glycocalyx disruption may have two principal consequences. First, compromised barrier integrity may facilitate the access of exogenous toxicants and endogenous tear components to epithelial receptors and cell membranes. Second, structural alterations of the glycocalyx may directly modify membrane-associated signaling by changing receptor accessibility, receptor organization, and cell–surface interactions, independent of its barrier function. Following impairment of the glycocalyx, toxicants may increasingly interact with lipid raft microdomains, which function as specialized signaling platforms regulating, for example, calcium signaling, redox signaling, inflammation, immune responses, and apoptosis (17).

#### 3.3.2 Integrated regulation of epithelial apoptosis

The second model proposes that epithelial apoptosis represents a central point of convergence linking the three discussed mechanisms. Environmental toxicants, hyperosmolarity, oxidative stress, inflammatory mediators, and metabolic disturbances may initially disturb glycocalyx integrity and activate lipid raft-mediated signaling. These membrane-associated events are known to regulate oxidative stress, calcium homeostasis, inflammatory pathways, and both intrinsic and extrinsic apoptotic signaling (9, 17, 69). In addition, formaldehyde exposure may reduce epithelial cell viability, induce chemokine production, promote apoptosis, and increase inflammation (116, 118, 119).

Apoptosis-associated tear miRNAs may subsequently modulate these downstream responses through post-transcriptional regulation of gene expression. Several tear miRNAs identified in DED have previously been associated with apoptosis in other biological systems (53–68), although their functional role at the ocular surface remains unknown. Through RISC-mediated post-transcriptional regulation of apoptosis-related target genes (69), tear miRNAs may either promote or suppress epithelial apoptosis depending on the specific genes and signaling pathways they regulate. Conversely, epithelial injury itself may alter extracellular vesicle secretion and tear miRNA composition, thereby creating bidirectional interactions between apoptosis and extracellular RNA signaling.

#### 3.3.3 Feed-forward amplification of ocular surface damage

The third model proposes that these mechanisms become interconnected through positive feedback loops that progressively amplify ocular surface injury. Initial glycocalyx disruption may facilitate greater penetration of environmental toxicants while simultaneously altering epithelial signaling. Enhanced lipid raft activation may subsequently amplify oxidative stress, inflammatory signaling, and apoptotic responses. These events may further alter extracellular miRNA release and intercellular communication, influencing neighboring epithelial cells.

At the same time, apoptosis, oxidative stress, inflammatory cytokines, and proteolytic activity may further compromise glycocalyx integrity through altered mucin expression, glycosylation, and degradation of glycocalyx components. In parallel, hapten formation following reactions between reactive chemicals such as formaldehyde and glycocalyx-associated proteins may generate neoantigens capable of sustaining allergic and inflammatory responses, thereby providing an additional mechanism for persistent epithelial injury. Lipid raft remodeling may further increase cellular responsiveness to inflammatory stimuli, creating additional feedback between membrane signaling and epithelial damage.

Collectively, the proposed models provide a conceptual framework for investigating how multiple underexplored mechanisms may converge to influence ocular surface homeostasis in DED. Although these interactions remain hypothetical, they generate experimentally testable hypotheses and may help guide future studies investigating the interplay between environmental stress, epithelial barrier function, membrane-associated signaling, and apoptosis. Future studies integrating controlled environmental exposure models, glycocalyx characterization, lipid raft imaging, extracellular vesicle profiling, and functional analyses of tear miRNAs will be required to determine the validity of these proposed interactions. To facilitate experimental validation of these hypotheses, **Table 2** summarizes key *in vitro*, animal, and clinical/observational approaches that could directly test the proposed mechanisms.

**Table 2:** Key experimental approaches for validation of the proposed mechanisms.

Proposed mechanism	*In vitro* validation	Animal validation	Clinical/observational validation
Tear miRNAs and apoptosis	Standardized tear collection and extracellular vesicle profiling; exposure of ocular surface epithelial models to patient-derived tear fractions or candidate miRNAs; miRNA mimic/inhibitor approaches with apoptosis and target validation	*In vivo* modulation of candidate miRNAs followed by assessment of ocular surface epithelial apoptosis and disease-related phenotypes	Longitudinal tear miRNA profiling correlated with DED severity, disease activity, and apoptosis-related biomarkers
Toxicant-induced glycocalyx alterations	Air–liquid interface ocular surface models with controlled nanoparticle or volatile-compound exposure; high-resolution imaging and molecular characterization of glycocalyx structure and function	Controlled ocular pollutant-exposure models assessing glycocalyx integrity, mucin/glycan changes, barrier function, and inflammatory responses	Assessment of glycocalyx/mucin-related biomarkers in relation to environmental exposure and DED characteristics
Toxicant-induced lipid raft disruption	Live-cell imaging and membrane fractionation to assess lipid raft dynamics and composition, integrated with controlled toxicant exposure and experimental modulation of membrane lipids	Ocular toxicant-exposure models assessing lipid raft organization, associated signaling, oxidative stress, apoptosis, and barrier function	Tear lipid or membrane-associated biomarker profiling in relation to environmental exposure and DED phenotypes

## 4 Therapeutic implications

Progress in these emerging areas remains constrained by several methodological and conceptual challenges, as outlined in the previous sections. Addressing these limitations will be essential for translating hypothesis-driven insights into experimentally testable and clinically relevant knowledge. In this context, elucidating the pathogenic mechanisms underlying DED may facilitate the future development of targeted therapeutic strategies. Beyond treatments primarily aimed at symptom relief and broad anti-inflammatory effects (22), emerging insights such as apoptosis-related pathways, tear miRNA dysregulation, and ocular surface structural changes provide potential directions for future mechanism-based interventions, although many of these concepts remain to be experimentally validated (6). In line with current clinical approaches, increasing emphasis is placed on therapeutic strategies that restore tear film stability and lacrimal and meibomian gland function as central components of ocular surface homeostasis (143). Such advances may help explain interindividual variability in treatment responses and provide a rational foundation for the development of novel therapies.

The potential of miRNAs as therapeutic agents for ocular surface and lacrimal gland diseases has previously been addressed (37). For DED, a 2022 study using rodent models showed that anti-miR-328 promoted corneal re-epithelialization, reduced apoptosis in corneal epithelial and stromal cells, and alleviated meibomian gland orifice obstruction (144). The same group earlier reported that the inhibition of miR-328 increased ocular mucin expression and conjunctival goblet cells (145). They also indicated that overexpression of miR-328 in the eye is a risk factor for myopia (146). A randomized phase II trial has been reported to evaluate the efficacy and safety of SHJ002, an anti-miRNA-328 eye drop formulation, for the treatment of DED (147). Liao et al. (144) suggested that miRNA-based therapy could represent an alternative treatment for DED patients who do not respond to conventional anti-inflammatory approaches. However, further molecular studies are needed to clarify the underlying mechanisms. Some attempts have also been made in Sjögren syndrome dry eye (148–151). Interestingly, antiapoptotic miRNAs have been characterized in Sjögren syndrome using cultured human submandibular gland cells and salivary gland tissues (152). Future work in this area would benefit from approaches that combine standardized tear sampling with extracellular vesicle profiling and functional validation in epithelial models, allowing direct assessment of miRNA-mediated apoptotic regulation.

Interactions among environmental pollutants, ocular allergies, and the glycocalyx of keratoconjunctival epithelial cells raise mechanistic considerations relevant to ocular surface health. As the glycocalyx represents the primary molecular interface between airborne contaminants and epithelial cells, pollutant-induced disruption of this layer may increase epithelial exposure, facilitate neoantigen formation, and promote immune activation at the ocular surface. Accordingly, strategies aimed at preserving glycocalyx structural and functional integrity may represent promising future therapeutic approaches to limit these processes, although their efficacy in DED remains to be established. In susceptible conditions such as DED, barrier-supportive approaches, including formulations designed to maintain mucin organization, hydration, or surface charge, may reduce vulnerability to pollution-associated ocular surface injury. In addition, emerging clinical perspectives emphasize the role of modifiable environmental and behavioral factors—such as digital device use and blinking dynamics—in disrupting tear film homeostasis, supporting the inclusion of nonpharmacological and lifestyle-oriented interventions within the broader therapeutic framework for DED (153). For instance, pharmacological agents such as rebamipide and diquafosol sodium address mucin deficiency in patients by enhancing mucin expression and secretion on the ocular surface (86).

Treatment with rebamipide ophthalmic suspension has been shown to significantly improve subjective symptoms, including ocular pain and foreign body sensation, which are often insufficiently relieved by conventional treatment. These effects are likely related to reduced eyelid–ocular surface friction resulting from MUC16 protein-mediated restoration of the glycocalyx barrier (10). The physicochemical properties of topical treatments also warrant careful consideration, as repeated exposure to certain excipients, preservatives, or particulate matter may influence glycocalyx structure in a manner analogous to airborne nanoparticles further affecting epithelial defense and ocular surface homeostasis. Advancing this field will require experimental systems that combine controlled pollutant exposure with high-resolution characterization of glycocalyx structure and function, enabling direct investigation of these interactions at the ocular surface.

Recognition of lipid rafts as functional regulators of ocular surface epithelial signaling suggests potential therapeutic opportunities aimed at preserving or restoring membrane microdomain integrity under e.g., environmental stress. However, given the currently limited and largely indirect mechanistic evidence, these considerations should be regarded as conceptual rather than immediately translatable strategies. Approaches such as lipid-based tear substitutes, formulations modulating cholesterol or sphingolipid content, and antioxidants protecting membrane lipids from oxidative damage, may represent hypothetical avenues to help maintain barrier function and attenuate pro-inflammatory signaling relevant to DED (23, 154, 155). At present, these strategies remain speculative and require direct experimental validation in ocular surface models. In addition, integrating tear fluid biomonitoring with raft-focused mechanistic analyses could, in principle, enable more personalized interventions by detecting exposure-related membrane perturbations and guiding preventive or adjunctive therapies. Further progress will depend on the application of approaches capable of probing lipid raft dynamics in living epithelial systems, including live-cell imaging and membrane fractionation techniques, ideally integrated with controlled toxicant exposure models, as these will be essential for testing and validating the proposed mechanism-based therapeutic concepts.

## 5 Conclusion and future directions

DED is a complex, multifactorial condition in which established mechanisms only partially explain disease onset and progression. This work highlights three emerging mechanisms that may contribute to the pathophysiology of DED and offers avenues for future research. First, apoptosis-related tear miRNAs, beyond their proposed role as biomarkers, may actively regulate epithelial cell death and influence inflammatory and regenerative responses at the ocular surface. Second, glycocalyx alterations, particularly those driven by environmental pollutants such as airborne nanoparticles and reactive volatile compounds, may compromise epithelial barrier function, facilitate neoantigen formation, and exacerbate ocular surface vulnerability. Third, lipid raft disruption represents a largely unexplored mechanism through which environmental stressors may perturb cellular signaling, apoptosis, and barrier integrity.

Despite their potential relevance, progress in these areas remains limited by several methodological and conceptual challenges. These include variability in tear sampling and analysis, limited availability of physiologically relevant ocular surface models, and technical constraints in studying extracellular miRNA function and nanoscale membrane structures such as the glycocalyx and lipid rafts under dynamic conditions. In addition, much of the current mechanistic understanding is derived from non-ocular systems, underscoring the need for ocular-specific validation.

Integrating these mechanisms provides a broader perspective on DED pathophysiology and underscores the need for mechanistic studies that go beyond symptom management. Future research should not only aim to further characterize these pathways but also to implement experimentally tractable strategies to address current limitations. In addition, it has to focus on elucidating the functional roles of tear miRNAs in apoptotic and damage-associated pathways, characterizing pollutant-glycocalyx interactions at a molecular level, and investigating how lipid raft integrity affects ocular surface resilience under environmental stress. In this context, approaches such as standardized tear collection protocols, extracellular vesicle profiling, air–liquid interface epithelial models, and high-resolution imaging or membrane fractionation techniques may provide practical routes to advance these fields. Additionally, exploring the interplay among these mechanisms may reveal synergistic or cumulative effects that contribute to disease heterogeneity.

From a therapeutic standpoint, mechanism-based interventions hold promise for personalized treatment strategies. Approaches that restore or stabilize miRNA profiles, protect glycocalyx structure and function, or preserve lipid raft organization could complement current anti-inflammatory and lubrication therapies. Furthermore, integrating tear fluid biomonitoring with these emerging mechanistic insights may enable early detection of environmental or molecular perturbations, thereby facilitating preventive strategies and optimizing therapeutic responses.

Ultimately, progress will depend on closer alignment between hypothesis-driven concepts and experimentally testable frameworks, enabling translation from conceptual models to mechanistic validation. In summary, advancing our understanding of apoptosis-related miRNAs, glycocalyx integrity, and lipid raft dynamics offers a path toward more comprehensive, targeted, and individualized management of DED. Continued interdisciplinary research combining molecular, cellular, and environmental approaches will be essential to translate these insights into effective diagnostics and therapies.

## Data Availability

The original contributions presented in the study are included in the article/supplementary material. Further inquiries can be directed to the corresponding author.
